# Oxidative stress dependent microRNA-34a activation via PI3Kα reduces the expression of sirtuin-1 and sirtuin-6 in epithelial cells

**DOI:** 10.1038/srep35871

**Published:** 2016-10-21

**Authors:** J. R. Baker, C. Vuppusetty, T. Colley, Andriana I. Papaioannou, P. Fenwick, Louise Donnelly, K. Ito, P. J. Barnes

**Affiliations:** 1 Airway Disease Section, National Heart and Lung Institute, Imperial College, London SW3 6LY, U.K; 2 3rd Respiratory Medicine Department, Sismanogleio Hospital, Marousi Athens Greece

## Abstract

Sirtuin-1 (SIRT1) and SIRT6, NAD^+^-dependent Class III protein deacetylases, are putative anti-aging enzymes, down-regulated in patients with chronic obstructive pulmonary disease (COPD), which is characterized by the accelerated ageing of the lung and associated with increased oxidative stress. Here, we show that oxidative stress (hydrogen peroxide) selectively elevates microRNA-34a (miR-34a) but not the related miR-34b/c, with concomitant reduction of SIRT1/-6 in bronchial epithelial cells (BEAS2B), which was also observed in peripheral lung samples from patients with COPD. Over-expression of a miR-34a mimic caused a significant reduction in both mRNA and protein of SIRT1/-6, whereas inhibition of miR-34a (antagomir) increased these sirtuins. Induction of miR-34a expression with H_2_O_2_ was phosphoinositide-3-kinase (PI3K) dependent as it was associated with PI3Kα activation as well as phosphatase and tensin homolog (PTEN) reduction. Importantly, miR-34a antagomirs increased SIRT1/-6 mRNA levels, whilst decreasing markers of cellular senescence in airway epithelial cells from COPD patients, suggesting that this process is reversible. Other sirtuin isoforms were not affected by miR-34a. Our data indicate that miR-34a is induced by oxidative stress via PI3K signaling, and orchestrates ageing responses under oxidative stress, therefore highlighting miR-34a as a new therapeutic target and biomarker in COPD and other oxidative stress-driven aging diseases.

Oxidative stress is a result of an imbalance between the production of free radicals and anti-oxidants, which detoxify or counteract the free-radicals’ harmful effects. It causes inflammation, damage of the cell membrane, protein modification (oxidation, carbonylation) and DNA damage^1^, and therefore, is suspected to be important in cardiovascular diseases, respiratory disease including asthma, chronic obstructive pulmonary disease (COPD) and cystic fibrosis, as well as rheumatoid arthritis, cancer and inflammatory bowel disease^2^,^3^,^4^. In addition, there is evidence to suggest free radicals are involved in the aging process and/or cellular senescence^5^. Particularly, the free radical aging theory is concerned with free radicals such as superoxide (O^2−^), hydrogen peroxide (H_2_O_2_) or peroxynitrite (OONO^−^), which are derived from different sources such as activated inflammatory cells and structural cells, cigarette smoke, air pollution and kitchen smoke^6^.

COPD is a chronic inflammatory lung disease, which is one of the leading causes of death and disability in the world and is now the third leading cause of death in high income countries^7^,^8^. The disease is progressive and affects mainly the elderly, being related to lung aging^9^. Chronic exposure to cigarette smoke and household air pollution are the major risk factors for the disease^10^. Once the disease is established, endogenous oxidative stress results from the release of reactive oxygen species (ROS) from inflammatory and structural cells of the lungs, enhanced further by impaired endogenous antioxidant defenses^1^,^2^. Therefore, in COPD patients the increased oxidative stress persists even after smoking cessation^11^. Cellular senescence and the inhibition of antioxidant genes are evident in COPD, which are known to be regulated by sirtuins^12^,^13^,^14^.

Sirtuins (SIRT) are Class III histone deacetylase (HDAC) enzymes that catalyze NAD^+^-dependent deacetylation and/or ADP-ribosylation of target proteins^15^, and are homologous to the yeast transcriptional repressor Sir2^16^. SIRT1, the most studied family member, is involved in the regulation of numerous biological processes, including inflammation, cellular senescence, DNA repair, genomic stability and autophagy; via the deacetylation of upstream regulatory proteins. SIRT1 deacetylates NF-κB, forkhead box class O (FOXO)-3, p21, p16, p53, Klotho, β-catenin/Wnt and histones, all of which contribute to the pathology of COPD^12^,^14^,^15^,^17^,^18^. P16 and p21, which are cyclin-dependent kinase inhibitor proteins and induce G1 stage cell cycle arrest^19^, are well-known markers of senescence and have been shown to be elevated in expression in cells taken from COPD patients^13^,^20^. In this regard, SIRT1 has been implicated in the regulation of both senescence and the expression of p16 and p21^14^. SIRT1 and SIRT6 are down-regulated in expression in the peripheral lungs of patients with COPD, and this is mimicked *in vitro* by oxidative stress^12^,^21^. The down-regulation of SIRT1 in patients with COPD has been attributed to post-translational modifications and proteasomal degradation^21^. However, it is well documented that a decrease in the mRNA levels of SIRT1 and SIRT6 is found in patients with COPD, with no proposed mechanism^12^,^22^.

Micro-RNAs (miRNAs) are small endogenous non-coding RNAs, which are typically 18–23 nucleotides in length, and regulate the expression of several target genes and may act as a link between different signaling pathways. Mature miRNAs bind to a target mRNAs at complementary sites within the 3′–untranslated region (3′-UTR), triggering the down-regulation and suppression of the target gene^23^. miRNAs have been extensively studied in relation to disease due to their importance in an array of biological processes; including aging, cell proliferation, and apoptosis^17^,^18^,^24^. Recent studies have examined the roles of miRNA in COPD^25^,^26^,^27^,^28^, with miR-34a being shown to be up-regulated in patients with severe COPD^29^. MiR-34a has been shown to be an important regulator of SIRT1 in colon epithelial, breast cancer and endothelial cells^30^,^31^,^32^. MiR-34a has also been recently linked with the down-regulation of SIRT6 when over-expressed in primary human keratinocytes^33^. As well as regulating the expression of the SIRT1 and SIRT6, miR-34a has been shown to directly regulate the expression of Protein phosphatase-1 nuclear targeting subunit (PNUTS)^24^; this protein is associated with ageing and regulates several pathways involved in accelerated aging, including the regulation of telomere length, DNA damage responses and cell cycle progression^24^,^34^.

Currently no link has been demonstrated between miR-34a and the decreased levels of SIRT1 and SIRT6 in patients with COPD. It is therefore hypothesized that elevated levels of miR-34a, found within the peripheral lung of patients with COPD, may regulate the expression of SIRT1 and SIRT6 under conditions of increased oxidative stress. Understanding whether miR-34a regulates the translation of SIRT1 and SIRT6 is crucial in understanding this miRNAs role in the pathology of COPD or oxidative stress driven aging diseases.

## Results

### SIRT1 and miR-34a expression in COPD lung

**Figure 1:**
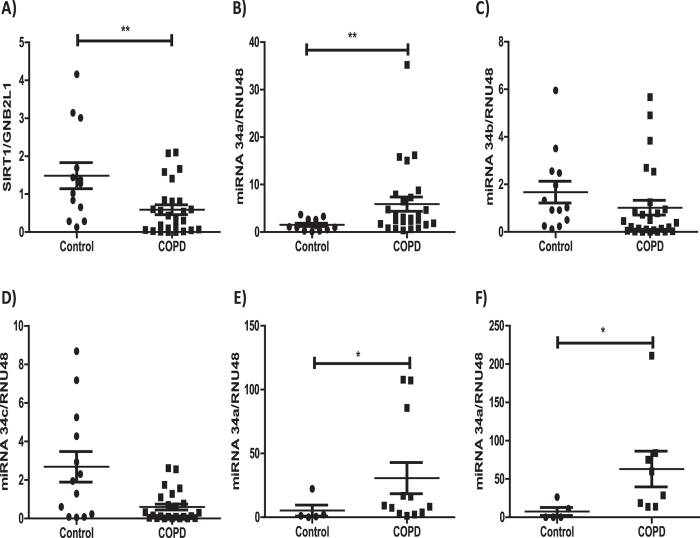
Decreased SIRT1 and increased miR-34a in COPD patients. Lung tissue from resections were obtained from 4 healthy volunteers and 9 non-COPD smoker volunteers (Controls), 15 mild COPD (Gold 1 plus Gold 2) and 11 severe COPD (Gold 3 plus Gold 4) (COPD) and RNA was extracted. **(A)** SIRT1 mRNA expression was examined in these lung samples and detected by QRT-PCR using a TaqMan assay normalized to GNB2L1 expression. **(B)** miR-34a levels, normalized to RNU48, were examine in lung samples from Control and COPD. **(C**,**D)** miR-34b and miR-34c levels, normalized to RNU48, were examined in lung samples from Control and COPD subjects. **(E)** miR-34a levels were examine in sputum cells samples from Control (N = 5) (1 non-smoker and 4 smokers) and COPD (N = 12) subjects. **(F)** miR-34a levels, normalized to RNU48, were examine in primary epithelial cells from Control (N = 5) (all non-smokers) and COPD (N = 7) subjects. Data are means ± SEM and analyzed by a Mann-Whitney U test * P < 0.05, **P < 0.01.

The expression of SIRT1 and miR-34a were examined in peripheral lung samples taken from control subjects (which included non-smokers and smokers) and COPD patients who had various stages of the disease (patient details described in Table S1). As previously reported^12^ SIRT1 mRNA, normalized to the expression of a house keeper gene (GNB2L1), was confirmed to be significantly down-regulated in the peripheral lungs of patients with COPD (Fig. 1A). In contrast, miR-34a was significantly up-regulated in patients with COPD compared to age-matched subjects without the disease (Fig. 1B). Within these samples there was a trend towards higher miR-34a expression with lower SIRT1 mRNA expression, but the correlation was not statistically significant (data not shown). MiR-34a gene expression also showed a trend towards an increase with age (data not shown), in agreement others previous finding in aged mice^24^. This induction seemed to be selective for miR-34a as the other two closely related miRNA-34 family members, miRNA-34b and miRNA-34c, showed a trend towards reduced expression in COPD (Fig. 1C,D). Both have previously been shown to be down-regulated in patients with COPD, with significance being associated with emphysema severity^35^. MiR-34a was also shown to be significantly up-regulated in sputum cell samples taken from a different cohort of COPD patients (Table S2, Fig. 1E) and in primary epithelial cells taken from COPD patients undergo lung revision surgery (Fig. 1F).

### Oxidative stress regulates expression of both miR-34a and SIRT1 in airway epithelial cells

**Figure 2:**
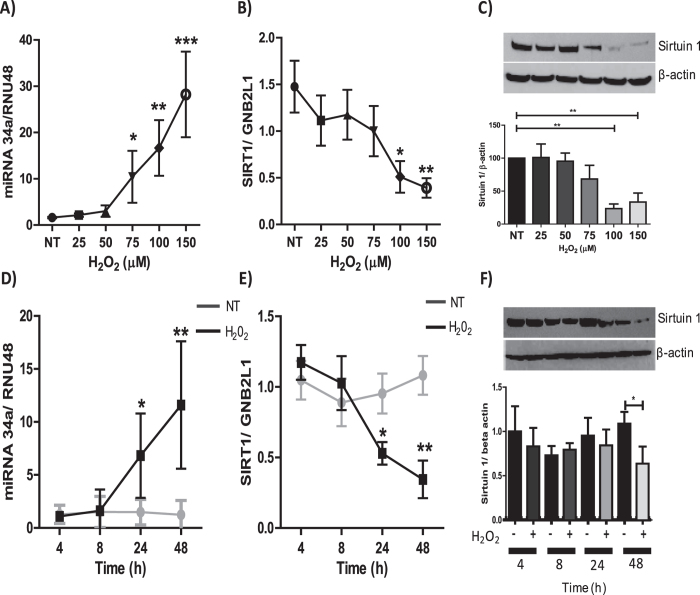
Correlation between oxidative stress-mediated reduction in SIRT1 and increased miR-34a expression. BEAS2B cells were stimulated for 48 hours with H_2_O_2_ at concentrations of 25, 50, 75, 100 and 150 μM, and protein or RNA extracted. **(A)** RNA was extracted to examine miR-34a (*n* = *6*) **(B)** and SIRT1 (*n* = *6*). **(C)** Protein was extracted and SIRT1 protein expression was determined by SDS-PAGE/Western blotting normalized to β-actin (*n* = *5*). BEAS2B cells were stimulated for 4, 8, 24 and 48 hours with 100 μM H_2_O_2_ and protein and RNA extracted, **(D)** changes in miR-34a expression was examined (*n* = *5*), as well as changes in **(E, F)** SIRT1 gene and protein expression (*n* = *5*). The band density of each blot is represented as a histogram and and is the average of all experiments performed. Data are means ± SEM, analyzed by Kruskal–Wallis test with post hoc Dunns and One-way Anova with post hoc Bonferroni *P < 0.05, **P < 0.01, ***P < 0.001.

To evaluate the effects of oxidative stress on both miR-34a and SIRT1 expression, immortalized human bronchial epithelial cells (BEAS2B) were treated for 48 hours with varying concentration of hydrogen peroxide (H_2_O_2_). A concentration-dependent increase in the levels of miR-34a was observed in cells treated with H_2_O_2_, with significant increases at concentrations 75, 100 and 150 μM (Fig. 2A). Conversely, both the mRNA and protein levels of SIRT1 were reduced at 100 and 150 μM of H_2_O_2_ (Fig. 2B,C). Time-course studies demonstrated that miR-34a induction was observed at 24 hours, indicating that miR-34a expression is induced prior to the maximal decrease of SIRT1 mRNA at 48 hours (Fig. 2D,E). SIRT1 protein was also decreased 48 hours after H_2_O_2_ treatment (Fig. 2F). These data suggest that oxidative stress modulates the expression of both SIRT1 and miR-34a in bronchial epithelial cells.

### MiR-34a directly regulates the expression of SIRT1

**Figure 3:**
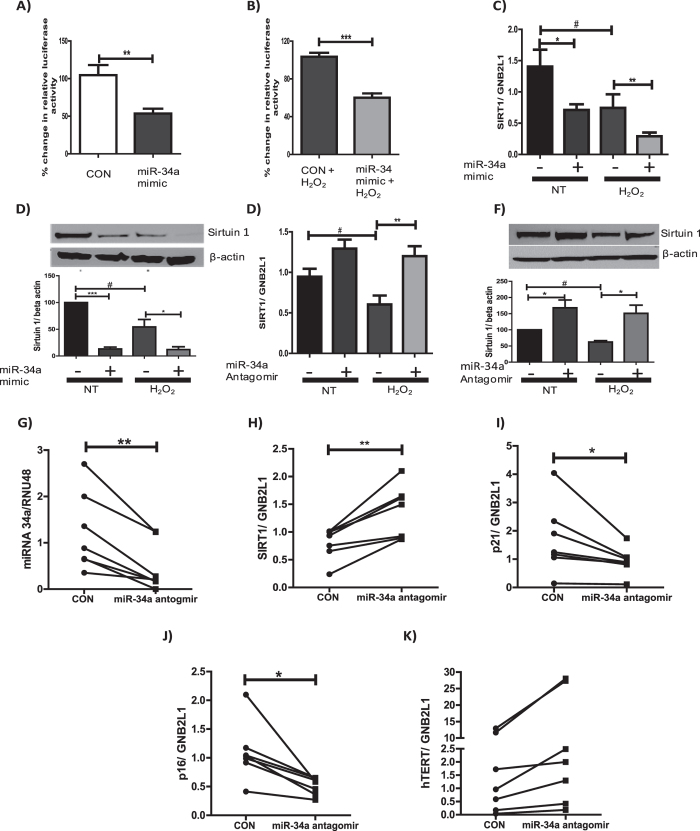
MiR-34a directly binds to SIRT1 mRNA 3′UTR and inhibits protein and mRNA expression. Luciferase assays were performed in BEAS2B cells. Co-transfection of a luciferase reporter with the 3′UTR of SIRT1 downstream of a luciferase gene (0.25 μg) and either a miR-34a mimic (15 nM) or control for 24 hours. **(A)** Cells were either left un-stimulated or **(B)** were stimulated for 48 hours with 100 μM H_2_O_2_ (*n* = *4*). **(C,D)** A miR-34a mimic or control were over-expressed for 24 hours and left un-treated or treated with 100 μM H_2_O_2_ for 48 hours and RNA or protein was extracted, SIRT1 gene and protein expression was assessed (*n* = *5*). BEAS2B cells were transfected with either a miR-34a antagomir (30 nM) or control and then left untreated or treated for 48 hours with 100 μM H_2_O_2_. RNA or protein was extracted and levels of SIRT1 **(E)** mRNA (*n* = *6*) or **(F)** protein (*n* = *4*) were assessed. Primary epithelial cells isolated from 7 COPD patients undergoing lung resection surgery were treated with miR-34a antagomirs (30 nM) for 24 hours. RNA was then extracted and **(G)** miR-34a, **(H)** SIRT1, **(I)** p21, **(J)** p16 or **(K)** hTERT mRNA levels were detected. The band density of each blot is represented as a histogram and is the average of all experiments performed. Data are means ± SEM analyzed by Mann-Whitney, Paired student t-test, Kruskal–Wallis test with post hoc Dunns and One-way Anova with post hoc Bonferroni *^#^P < 0.05, **P < 0.01, ***P < 0.001.

To assess whether miR-34a was directly regulating the expression of SIRT1 in bronchial epithelial cells luciferase reporter experiments were performed, assessing whether miR-34a directly bound to the 3′UTR of SIRT1. A luciferase reporter plasmid with the 3′UTR of SIRT1 cloned downstream of the luciferase gene was transfected into BEAS2B cells. At the same time, a double-stranded RNA mimic of miR-34a was over-expressed. In both the presence and absence of oxidative stress, over-expression of a miR-34a mimic significantly reduced luciferase activity, suggesting that miR-34a directly binds to the 3′ UTR of SIRT1 mRNA under normal and oxidative conditions (Fig. 3A,B). In addition, over-expression of a miR-34a mimic led to a decrease in both the mRNA and protein levels of SIRT1 under normal and oxidative stress (Fig. 3C,D), suggesting a direct causal link between increased miR-34a and decreased SIRT1.

### Inhibition of miR-34a restores SIRT1 expression to pre-oxidative stress levels

To assess whether the effects of oxidative stress on the expression of SIRT1 could be prevented, a miR-34a antagomir was over-expressed in BEAS2B cells. Over-expression led to increased SIRT1 mRNA expression under non-oxidative conditions, but this was not significant. Under conditions of oxidative stress (H_2_O_2_ 100 μM), where SIRT1 mRNA levels are decreased, over-expressing the miR-34a antagomir restored the level of SIRT1 mRNA and protein to baseline conditions (Fig. 3E,F).

MiR-34a antagomirs were also over-expressed in primary epithelial cells from COPD patients. Over-expression of the antagomir significantly decreased miR-34a expression, with this decrease leading to a significant increase in the mRNA expression of SIRT1 (Fig. 3G,H). These data suggested that miRNA antagomirs have the ability to rescue the loss of SIRT1 mRNA in COPD cells. The mRNA expression of p16 and p21 were also examined after the over-expression of miR-34a antagomirs. The expression of both of these markers of senescence was decreased after treatment (Fig. 3I,J), suggesting miR-34a antagomirs to prevent the induction of senescence in these cells. Similarly, the expression of hTERT, a key component of telomerase was examined, although not significant, an increase in hTERT expression was observed (Fig. 3K).

### MiR-34a also regulates the expression of SIRT6

**Figure 4:**
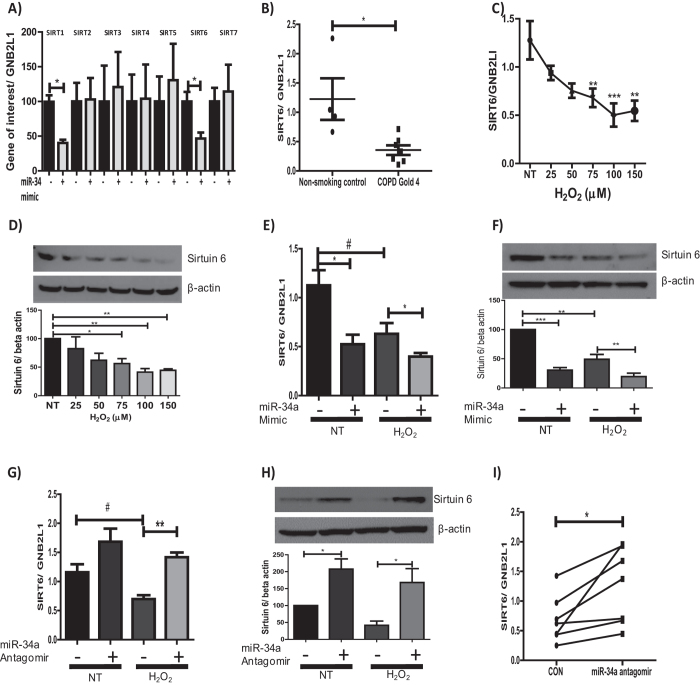
MiR-34a reduces the protein and mRNA expression of SIRT6. **(A)** A miR-34a mimic or control was over-expressed for 24 hours and the expression of SIRT1, 2, 3, 4, 5, 6 and 7 assessed (*n* = *4–7*). **(B)** Lung tissue from resections were obtained from 4 healthy volunteers and 7 COPD Gold stage 4 and RNA extracted, SIRT6 mRNA levels then examined. **(C)** BEAS2B cells stimulated for 48 hours with H_2_O_2_ at concentrations of 25, 50, 75, 100 and 150 μM and protein or RNA extracted and changes in SIRT6 gene expression examined (*n* = *5*). **(D)** SIRT6 protein expression was also assessed (*n* = *5*). **(E**,**F)** A miR-34a mimic (15 nM) or control was over-expressed for 24 hours and left un-treated or treated with 100 μM H_2_O_2_ for 48 hours. RNA or protein was extracted, SIRT6 gene and protein expression assessed (*n* = *5*). A miR-34a antagomir (30 nM) or control was over-expressed for 24 hours and left untreated or treated for 48 hours with 100 μM H_2_O_2_. RNA or protein was extracted and levels of SIRT6 **(G)** mRNA (*n* = *6*) or **(H)** protein were assessed (*n* = *3–5*). **(I)** Primary bronchial epithelial cells taken from 7 patients with COPD were transfected with either a miR-34a antagomir (30 nM) or control for 24 hours. RNA was extracted and SIRT6 mRNA were detected (*n* = *7*). The band density of each blot is represented as a histogram and is the average of all experiments performed. Data are means ± SEM analyzed by Mann-Whitney, Paired student t-test, Kruskal–Wallis test with post hoc Dunns and One-way Anova with post hoc Bonferroni *P < 0.05, **P < 0.01, ***P < 0.001.

Along with SIRT1, SIRT6 is the only other sirtuin isoform down-regulated in patients with COPD^12^. MiR-34a has also been shown to directly regulate SIRT6 expression by binding to the 3′UTR of SIRT6 mRNA, decreasing both the mRNA and protein expression^33^. To assess whether miR-34a regulates the expression of this isoform or any other members of the sirtuin family in epithelial cells, a miR-34a mimic was over-expressed and the mRNA expression of each sirtuin isoform assessed. Data showed that SIRT1 and SIRT6 were the only members of the sirtuin family down-regulated by the miR-34a mimic, with the mRNA expression of SIRT2, 3, 4, 5 and -7 being unaffected by the mimic (Fig. 4A), implying the importance of miR-34a in COPD.

SIRT6 expression was examined in our peripheral lung sample cohort, with data showing SIRT6 to be down-regulated at the mRNA level in COPD patients, but only when comparing non-smoking controls to patients with the most severe form of COPD (GOLD stage 4) (Fig. 4B). To assess whether oxidative stress was involved in the down-regulation of SIRT6, BEAS2B cells were treated with increasing concentrations of H_2_O_2_ and the mRNA and protein levels of SIRT6 examined. At the higher concentrations of H_2_O_2_ (75, 100 and 150 μM) a significant decrease in the expression of SIRT6 was seen at both the mRNA and protein level (Fig. 4C,D). Over-expression of a miR-34a mimic led to the suppression of both the mRNA and protein expression of SIRT6 in epithelial cells under normal and oxidative conditions (Fig. 4E,F). When an antagomir of miR-34a was over-expressed, under conditions of oxidative stress, the expression of SIRT6 mRNA and protein was restored to pre-oxidative conditions (Fig. 4G,H). An antagomir of miR-34a over-expressed in bronchial epithelial cells taken from patients with COPD also led to a significant increase in the mRNA expression of SIRT6, suggesting the reduction of SIRT6 is reversible (Fig. 4I). These data, as similarly seen for SIRT1, suggest that miRNA antagomirs have the capability to restore the loss of SIRT6 mRNA in patients with COPD.

### Oxidative stress modulates miR-34a expression via the PI3K pathway

**Figure 5:**
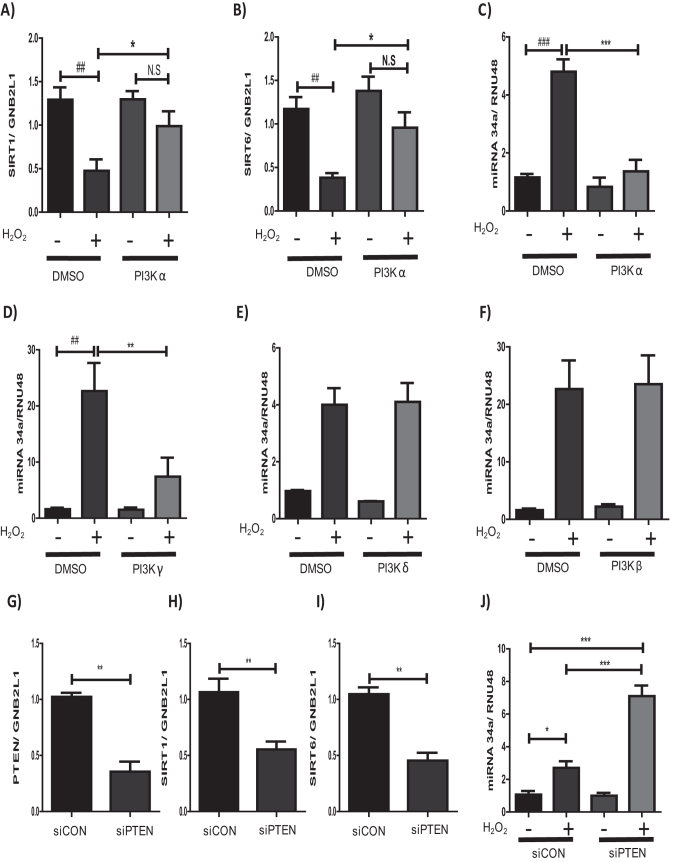
Pathways through which oxidative stress may regulate miR-34a expression. BEAS2B cells were treated with either PIK75 (at 10 μM) or vehicle (DMSO) for 1 hour prior to stimulation with or without 100 μM H_2_O_2_ for 48 hours, RNA extracted and **(A)** SIRT1 or **(B)** SIRT6 expression examined (*n* = *6*). BEAS2B cells were treated with either **(C)** PIK75, **(D)** AS-605240, **(E)** IC-87114 and **(F)** GSK2636771 (10 μM) or vehicle (DMSO) for 1 hour prior to stimulation with or without 100 μM H_2_O_2_ for 48 hours, RNA extracted and miR-34a levels assessed (*n* = *3–5*). BEAS-2B cells were transfected with small interfering RNA (siRNA) against either PTEN for 24 h or a random oligonucleotide control and then either left un-stimulated or stimulated with 100 μM H_2_O_2_ for 48 hours (*n* = *4*). RNA was extracted and either **(G)** PTEN, **(H)** SIRT1, **(I)** SIRT6 or **(J)** miR-34a levels assessed. Data are means ± SEM analyzed by Mann-Whitney and Kruskal–Wallis test with post hoc Dunns ^#^P < 0.05, *P < 0.05, ***P < 0.001.

To understand the potential mechanisms by which oxidative stress elevates the levels of miR-34a, the role of the PI3K signaling pathway was examined. This pathway has previously been implicated the regulation of SIRT1 via oxidative stress^36^ and is a crucial signaling pathway in the pathogenesis of COPD^37^. As PI3Kα is the dominant isoform expressed in bronchial epithelial cells^35^ PI3Kα was inhibited using PIK75, a PI3Kα selective inhibitor. PI3Kα inhibition led to a significant increase in the expression of both SIRT1 and SIRT6 in cells treated with H_2_O_2_ compared to vehicle control (Fig. 5A,B), showing the importance of this pathway in the reduction of SIRT1 and SIRT6 under conditions of oxidative stress.

To further understand the mechanism by which the PI3K pathway regulates both SIRT1 and SIRT6, we examined the effect of PI3K inhibition on miR-34a expression, using 4 isoform specific PI3K inhibitors. H_2_O_2_-induced miR-34a expression in epithelial cells was significantly abrogated by PI3Kα (PIK75) and PI3Kγ inhibition (AS-605240), but not when PI3Kβ (GSK2636771) or PI3Kδ (IC-87114) were inhibited (Fig. 5C–F). When phosphatase and tensin homolog (PTEN), a negative regulator of PI3K signaling was knocked-down (Fig. 5G) under oxidative conditions, a significant decrease in SIRT1 and SIRT6 mRNA expression was seen (Fig. 5H,I). In addition to the loss of SIRT1 and SIRT6, an induction of miR-34a was seen (Fig. 5J), further implicating the dysregulation of the PI3K pathway in the expression of miR-34a, SIRT1 and SIRT6 in COPD.

### MiR-34a regulates PNUTS, an age associated driver of the accelerated ageing phenotype in COPD patients

**Figure 6:**
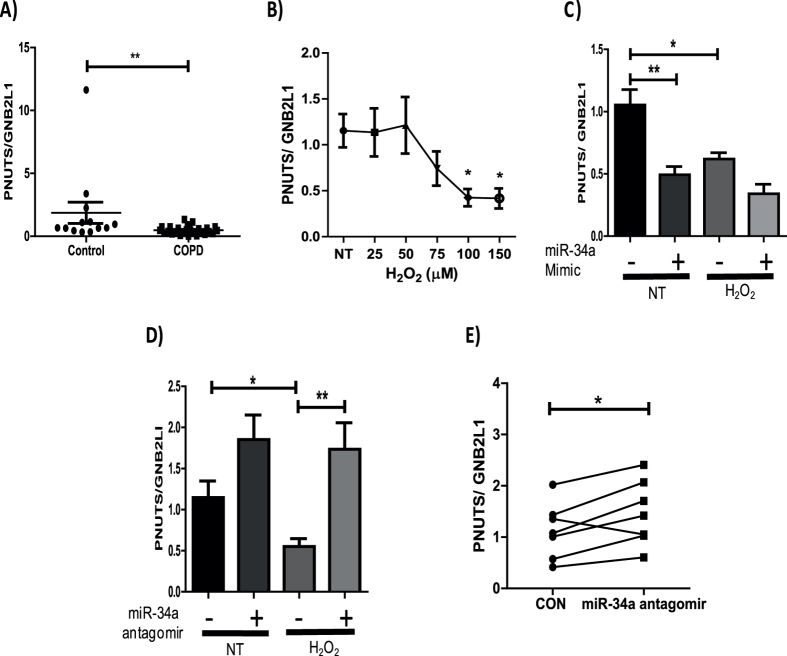
MiR-34a regulates the expression of the age associated protein, PNUTS. **(A)** RNA was extracted from lung resection tissue and PNUTS mRNA levels examined. **(B)** BEAS2B cells were stimulated for 48 hours with H_2_O_2_ at concentrations of 25, 50, 75, 100 and 150 μM and RNA extracted. PNUTS gene expression was assessed (*n* = *4*). **(C)** A miR-34a mimic or control were over-expressed in BEAS2B cells for 24 hours and left un-treated or treated with 100 μM H_2_O_2_ for 48 hours. RNA or protein was extracted and PNUTS gene expression assessed. (**D)** BEAS2B cells were transfected with either a miR-34a antagomir (30 nM) or control and either left untreated or treated for 48 hours with 100 μM H_2_O_2_. RNA was extracted and PNUTS mRNA expression assessed (*n* = *8*). (**E**) Primary bronchial epithelial cells taken from 7 patients with COPD were transfected with either a miR-34a antagomir (30 nM) or RNA control for 24 hours. RNA was then extracted and PNUTS mRNA levels were detected (*n* = *7*) Data are means ± SEM analyzed by Mann-Whitney, Paired student t-test, Kruskal–Wallis test with post hoc Dunns and One-way Anova with post hoc Bonferroni *P < 0.05, **P < 0.01.

PNUTS (also known as PPP1R10), a direct target of miRNA-34a, has been shown to be down-regulated with age as well as being associated with the regulation of telomere length, DNA damage responses and apoptosis^24^, all of which are deregulated in COPD. The role of PNUTS in COPD pathogenesis has not been examined, with no previous studies investigating the expression of this phosphatase in COPD patients. When examining the mRNA expression of PNUTS, in our peripheral lung samples, a significant decrease in expression was seen in COPD patients (Fig. 6A), with the decrease being observed in patients with the most severe form of the disease (Figure S1). The effects of oxidative stress on the mRNA expression of PNUTS was examined, showing PNUTS gene expression to be significantly down-regulated in epithelial cells treated with H_2_O_2_ (100 and 150 μM) (Fig. 6B). To assess direct regulation of PNUTS by miR-34a, a miR-34a mimic was over-expressed. Over-expression induced a significant decrease in the mRNA expression of PNUTS at basal levels and a further reduction in expression was seen in conditions of oxidative stress, although this was not significant (Fig. 6C). Over-expression of a miR-34a antagomir rescued the loss of PNUTS mRNA expression to above basal levels when treated with H_2_O_2_ (Fig. 6D). An antagomir of miR-34a was also over-expressed in bronchial epithelial cells taken from patients with COPD. This led to a significant increase in the mRNA expression of PNUTS, suggesting the reduction of PNUTS in COPD patients could be reversible (Fig. 6E). These data, as similarly seen for SIRT1 and SIRT6, suggest that miRNA antagomirs have the capability to restore the loss of PNUTS mRNA in patients with COPD.

## Discussion

SIRT1 and SIRT6 are putative anti-ageing molecules that have been shown to be significantly reduced in the lungs of COPD patients, in agreement with the view that COPD represents acceleration of lung aging^12^,^23^. They have both been shown to down-regulate inflammation through the modulation of NF-κB signaling, reduce emphysema through inhibition of matrix metalloproteinase expression, and also regulate the expression of several anti-oxidant genes^12^,^14^,^38^,^39^. These findings have shown the importance of these two deacteylases in the pathology of COPD. Oxidative stress, a key feature of COPD, is believed to be a main driver of the down-regulation of SIRT1 and SIRT6 protein expression in COPD patients^12^,^21^,^39^. However, the mechanism by which the mRNA levels of these deacetylases are down regulated in COPD has not been understood until now.

MiRNAs have been extensively documented in COPD, with many miRNAs being associated with disease severity and clinical phenotypes^27^,^28^,^40^. However, much of this work has focused on alterations in expression between healthy and COPD patients, with little information on how the dysregulation of these miRNAs contribute to disease mechanisms^35^,^41^. Although miR-34a has been previously been shown to be up-regulated in the lungs of patients with COPD^29^, here, we show for the first time that abnormal regulation of miR-34a by oxidative stress causes parallel down-regulation of SIRT1 and SIRT6.

MiR-34a is known to regulate SIRT1 expression^30^, but this has not been examined in the context of COPD. Elevated levels of this miRNA were seen in lung homogenates from COPD patients in association with reduced expression of SIRT1. MiR-34a was also elevated in sputum and primary bronchial epithelial cells taken from COPD patients, suggesting increased miR-34a expression in a heterogeneous population of cells. To explore the specificity of these findings the expression of the two other members of the miRNA-34 family, miR-34b and miR-34c, were examined. These two miRNAs have previously been shown to be down-regulated in COPD patients^35^ and we confirmed that both were reduced in COPD lung parenchyma, although this did not quite achieve statistical significance^35^. When, however, the control samples were further analyzed, separating smokers and non-smokers, a significant decrease in the expression of both miR-34b and miR-34c is seen (Figures S2 and S3). These data suggest smoking to regulate their expression, this however was not observed for miR-34a (Figure S4). One explanation for the decreased expression of these two miRNAs could be the reduced expression of FOXO3a in COPD^42^. This transcription factor has been shown to regulate the expression of both these miRNAs^43^, but we found that FOXO3a silencing had no effect on the expression of miR-34a (Figure S5).

Increased oxidative stress is a key mechanism driving COPD pathogenesis, with elevated levels of oxidative stress persisting after smoking cessation^11^. Oxidative stress causes lung injury by inducing the depletion of glutathione (and other antioxidants), increasing and activating proteinases; all of which further perpetuate the levels of oxidative stress as well as inducing damage to lipids, nucleic acids and proteins^44^. However, the role of oxidative stress in the regulation of miRNAs has not previously been examined in COPD. Elevated oxidative stress decreased both the protein and mRNA levels of SIRT1, whilst up-regulating the expression of miR-34a. Interestingly, the induction of miR-34a began at between 8–24 hours after treatment, prior to the maximal decrease in SIRT1 mRNA and protein expression. This correlation led us to believe that oxidative stress may be inducing miR-34a, leading to a decrease in SIRT1, via the binding of miR-34a to SIRT1 mRNA, thereby inhibiting its translation and/or decreasing mRNA stability of SIRT1, resulting in its degradation.

MiR-34a directly binds to the 3′UTR of SIRT1 mRNA^30^, inducing a decrease in the protein and/or mRNA expression of this gene; this is via translation repression but may also be due to increased mRNA decay. Our data show that in bronchial epithelial cells, miR-34a directly binds to the 3′UTR of SIRT1 mRNA, in both the absence and presence of oxidative stress, suggesting a direct regulatory effect. As the regulation of SIRT1 by miR-34a has been shown to decrease just the protein levels of SIRT1 in certain cell types^30^,^31^, but also the mRNA and protein levels of SIRT1 in other cell types^45^, we assessed the regulation of both in bronchial epithelial cells. Over-expression studies showed miR-34a regulated both mRNA and protein expression of SIRT1, with the same results also being observed for SIRT6. These data imply a new mechanism by which the mRNA and protein levels of both SIRT1 and SIRT6 are decreased in parallel in patients with COPD.

As miR-34a has only been shown previously to regulate the expression of SIRT1 and SIRT6^30^,^33^, the only two members of the sirtuin family to be down-regulated in COPD^12^, we wanted to assess whether miR-34a was specific against just these two isoforms or the rest of the family. We first examined multiple miRNA target prediction websites; two target prediction websites miRanda and miRTarBase predicted only SIRT7 to also be a target of miR-34a. To further validate this we over-expressed a miR-34a mimic and assessed these effects on the mRNA expression of SIRT2, 3, 4, 5 and −7. Over-expression of miR-34a mimics had no effect on the expression of either of these family members, including SIRT7. These data suggest not only the specificity of this mimic, but also the importance of miR-34a in regulating only the sirtuin isoforms that are down-regulated in COPD, showing the importance of this miRNA in the disease.

To investigate whether the decrease in expression of SIRT1 and SIRT6 under conditions of oxidative stress could be prevented, miR-34a was inhibited in the presence of H_2_O_2_. Results showed both the mRNA and protein levels of SIRT1 and SIRT6 to be down-regulated in conditions of oxidative stress, but this decrease was completely prevented when miR-34a was inhibited by a specific antagomir. These findings were further validated when examining the effects of the miR-34a antagomir in COPD epithelial cells, which are known to have decreased sirtuin-1 and sirtuin-6 expression. A significant increase in the mRNA expression of SIRT1 and SIRT6 was seen in these primary epithelial cells when miR-34a was suppressed using the antagomir. These data imply that the reduction in SIRT1 and SIRT6 mRNA levels could be restored in COPD patients, suggesting administration of these antagomirs as a potential therapy to reverse the accelerated aging phenotype of COPD patients.

P21 and p16 are two important cyclin-dependent kinase inhibitors, which mediate cellular senescence^19^ and are associated with the ageing phenotype in COPD patients^46^. Over-expression of the miR-34a antagomir in COPD primary epithelial cells led to a significant decrease in the expression of p21 and p16. The mechanism by which miR-34a may be regulating the expression of p21 and p16 is not fully elucidated. However, SIRT1 has been previously shown to decrease the expression of p21^30^ in a miR-34a-dependent manner and SIRT1 is known to regulate both p21 and p16 expression under oxidative conditions^14^. In addition to examining the effects of the antagomir on these senescence markers we examined the antaogmirs effect on hTERT expression, a key component of telomerase. MiR-34a has previously been shown to regulate the expression of this protein, inducing cellular senescence^47^. Although not significant, an increase in the expression of hTERT is seen, suggesting a further mechanism by which miR-34a may be inducing cellular senescence. These data suggest miR-34a may act as a master regulator of the accelerating ageing phenotype, driving three distinct pathways, all of which can lead to accelerated ageing of the lung.

Previous studies of the signaling pathways regulating miR-34a expression have implicated p53 and NF-κB signaling, with both of these transcription factors being shown to bind directly to the promoter of miR-34a^48^,^49^. NF-κB signaling is up-regulated in patients with COPD^15^ and plays an important role in the up-regulation of inflammatory mediators seen within disease^50^. p53 is also up-regulated in patients with COPD and correlates with increased miR-34a expression^29^,^51^. However, in p53 knock-out mice the high expression of miR-34a in lungs persists, suggesting that p53-independent mechanisms may also regulate miR-34a transcription in the lung^52^. We therefore sought to understand whether further upstream mechanisms, known to be induced by oxidative stress and associated with COPD, were involved in the induction of miR-34a.

The PI3K signaling pathway is an important signaling pathway in the progression of COPD, being implicated in corticosteroid insensitivity in COPD patients and also being shown to be activated by oxidative stress^37^,^53^. To assess the role of this pathway in the regulation of SIRT1, SIRT6 and miR-34a various PI3K inhibitors were utilized. Inhibition of PI3Kα, the predominant isoform in bronchial epithelial cells, led to a significant increase in the expression of both SIRT1 and SIRT6 compared to controls when under conditions of oxidative stress. To understand the potential mechanism by which PI3K signaling modulated SIRT1 and SIRT6 expression the effect of isoform specific inhibition of the PI3K pathway on the expression of miR-34a was examined. Inhibition of PI3Kα led to a significant decrease in the expression of miR-34a, suggesting that activation of PI3K signaling by oxidative stress may induce miR-34a expression in patients with COPD. Interestingly, PI3Kγ, but not PI3Kδ or PI3Kβ, also appears to be involved in the regulation of miR-34a in epithelial cells. However, this inhibitor is believed to inhibit PI3Kα at the concentration used^54^ and may therefore suggest that only PI3Kα is regulating miR-34a expression in these conditions. The role of PTEN was also examined, as previously unpublished work by our group has shown this protein to be down-regulated in COPD patients. Knock-down of PTEN, the major endogenous inhibitor of PI3K signaling resulted in a significant increase in the levels of miR-34a and concomitant decrease in the mRNA expression of SIRT1 and SIRT6, further demonstrating the importance of the PI3K pathway in the regulation of miR-34a by oxidative stress. How PI3Kα and PI3K–γ isoforms regulate the expression of miR-34a remains unclear. However, PI3K signaling is known to induce NF-κB signaling via Akt^55^, suggesting that PI3K signaling may activate this transcription factor leading to its binding to the miR-34a promoter, thereby inducing miR-34a expression in bronchial epithelial cells.

As well as regulating the expression of SIRT1 and SIRT6, miR-34a has been shown to directly inhibit the expression of PNUTS^24^. This age associated protein has been shown to be important in the regulation of telomere length, apoptosis and DNA damage repair, all of which are deregulated in COPD. We therefore hypothesized that this protein might be down-regulated in patients in COPD, via the induction of miR-34a, through its induction by elevated oxidative stress. A decrease in the mRNA expression of PNUTS was seen in peripheral lung samples taken from COPD patients, with further data suggesting that the mRNA levels of PNUTS could be decreased by oxidative stress in BEAS2B cells. MiR-34a mimic and antagomir studies also confirmed the direct regulation of this protein by miR-34a. The over-expression of a miR-34a antagomir rescued the mRNA levels of PNUTS in epithelial cells from diseased patients, suggesting the reduction in the expression of this gene could be reversible in patients with COPD. The loss of PNUTS, via miR-34a, may induce apoptosis, cell cycle suppression and also senescence in COPD patients.

This study shows miR-34a to directly and specifically regulate the expression of SIRT1 and SIRT6 in bronchial epithelial cells. These data suggest a potential new mechanism by which the elevated levels of oxidative stress found within the peripheral lungs of patients with COPD decreases both the mRNA and protein expression of SIRT1 and SIRT6, as observed in patients with COPD. Over-expression of a miR-34a antagomir in bronchial epithelial cells from COPD patients suggests the decrease in SIRT1 and SIRT6 mRNA levels can be restored, preventing any further loss of these putative anti-ageing molecules. We also show PNUTS to be down-regulated in COPD patients and that miR-34a directly regulates the expression of this protein. These data suggest miR-34a may act as a master regulator in the expression of several anti-ageing molecules, showing the importance of this miRNA in the progression of COPD. MiR-34a antagomirs may therefore have the potential to be a new therapy to prevent further down-regulation of these anti-ageing molecules, slowing down the accelerated ageing phenotype of the lung and the loss of lung function in COPD patients. Finally, miR-34a may be a potential biomarker of cellular senescence in COPD.

## Materials and Methods

### Reagents and antibodies

H_2_O_2_ (Hydrogen peroxide) and 3-(4,5- dimethylthiazol-2 yr)-2-5-diphenyl tetrazoliumbromide (MTT) were purchased from Sigma (Poole, UK). PI3K inhibitors PIK75 hydrochloride (PI3K α) was purchased from Abcam (Cambridge, UK), GSK2636771 (PI3K β), AS-605240 (PI3K γ) and IC-87114 (PI3Kδ) were all purchased fromVWR International Ltd. (Leicestershire, UK). Antibodies against the following were used for immunoblotting: β-actin (Santa Cruz Biotechnology, Santa Cruz, CA), Sirtuin 1 (Epitomics, Cambridge, UK), Sirtuin 6 and Sirtuin 2 (Cell Signaling Biotechnology Beverly, MA, USA). Anti-rabbit (P0448) and anti-mouse (P0260) secondary antibodies were from Dako (Cambridge shire, UK Liopofectamin RNAimax and lipofectamine LTX plus were both purchased from (ThermoFischer, Massachusetts, USA).

### Cell culture and transfections

BEAS2B cells (human airway epithelial) (ATCC Teddington, UK) were cultured in keratinocyte media (Invitrogen, Paisley, UK) containing human recombinant epithelial growth factor (EGF) and bovine pituitary extracts (BPE). Human primary bronchial epithelial cells were cultured as monolayers in LHC-9 media (Invitrogen, Paisley, UK) on collagen (1% w/v) coated plates. Cells were extracted from lung tissue from patients undergoing lung resection surgery at the Royal Brompton Hospital. The subjects were matched for age and smokers and COPD patients for smoking history (Table S1). All subjects gave informed written consent and the study was approved by the NRES London-Chelsea Research Ethics committee, study number 09/H0801/85. All methods were performed in accordance with the relevant guidelines and regulations. All cells were serum starved 16 h before stimulation. Cells were stimulated with varying concentrations of H_2_O_2_ for time points indicated. BEAS2B and primary bronchial epithelial cells were transfected with mirVana miRNA mimics (mirVana™ miRNA Mimic Negative Control #1, has-miR-34a MC11030,) and inhibitors (mirVana™ miRNA Inhibitor Negative Control #1, has-miR-34a MH11030) (30 or 60 nM) (Ambion, Life Technologies, Foster City, CA) using Lipofectamine RNAimax for 24 or 48 hours prior to stimulation with H_2_O_2_. BEAS2B cells were transfected with siRNA (PTEN # 6251 and FOXO3a #6303 (Cell Signaling Technology), Negative control #1 (Ambion Silencer Select siRNA) (100nM) using Lipofectamine RNAimax for 24 hours prior to stimulation with H_2_O_2_.

### Lung tissue

COPD severity was graded according to the Global Initiative for Chronic Obstructive Lung Disease (GOLD) guidelines^56^ with lung function and symptoms. Lung tissues were obtained from an established tissue bank linked to an established patient registry which has previously been used^57^. mRNA and miRNAs were extracted using the miRNeasy kit (Qiagen) according to the manufacturer’s instructions.

### Sputum samples

Sputum was induced using 3% (weight/volume) nebulized hypertonic saline. Saliva was removed from the sputum samples and then protein and RNA was extracted using the mirVana PARIS RNA and Native Protein Purification Kit, as instructed by the manufacturer’s instructions. RNA samples were then reverse transcribed as previously stated and qPCR performed. Sputum samples were a kind gift from Andriana I Papaioannou and written informed consent was acquired.

### RNA extraction and Real-Time quantitative PCR

mRNA and miRNAs were extracted using the miRNeasy kit (Qiagen) according to the manufacturer’s instructions. RNAs were then reverse-transcribed using the TaqMan normal RNA and MicroRNA Reverse Transcription Kit (Life Technologies). Both normal and miRNA levels were detected by either TaqMan Assays (SIRT1 Hs01009006, SIRT2 Hs00247263, SIRT3 Hs00953477, SIRT4 Hs00202033, SIRT5 Hs00978335. SIRT6 Hs0021303, SIRT7 Hs01034735, GNB2L1 Hs00272002, and PTEN Hs02621230, PNUTS Hs00160391, p21 Hs00355782, p16 Hs00923894), or TaqMan MicroRNA Assay (hsa-miR-34a-5p 000426, has-miR-34b-5p 000427, has-miR-34c-5p 000428) (Applied Biosystems, Life Technologies, Foster City, CA). RNU 48 (001006), a small noncoding RNA, was detected as the endogenous control for miRNA detection and guanine nucleotide binding protein -polypeptide 2-like 1 (GNB2L1), as endogenous control for normal cDNA. After the reactions, the CT values were determined using fixed-threshold settings.

### Luciferase assay

Briefly, the day before transfection, cells were seeded onto 24-well plates. After 24 hours, 0.2 μg of Luc-SIRT1 3′UTR (Luc-SIRT1 3′UTR was a gift from Charles Lowenstein (Addgene plasmid #20379), 0.1 μg of renilla expression vector, were transfected into cells for 24 hours using Lipofectamine LTX plus reagent. These were co-transfected with and 30 nM of mirVana mimics or control. Cells were then serum starved for 16 h and stimulated with H_2_0_2_ (100 μM) for 48 hours. Dual-luciferase assay was conducted using Dual-Luciferase Reporter Assay System (Promega, Madison, WI, USA), with changes in firefly luciferase expression being normalized to renilla expression.

### Western Blotting

Protein extracts were prepared using RIPA buffer (Sigma: 150 mM NaCl, 1.0% IGEPAL® CA-630, 0.5% sodium deoxycholate, 0.1% SDS, and 50 mM Tris, pH 8.0.) completed with protease (Roche, Welwyn Garden City, UK). Protein extracts (40 μg) were analyzed by SDS-PAGE (Invitrogen, Paisley, UK) and detected with Western Blot analysis by chemiluminescence (ECL Plus; GE Healthcare, Hatfield, UK). Protein expression levels were expressed relative to β-actin.

### Statistical Analysis

Data are expressed as SEM. Results were analyzed using t-test and one- or two-way ANOVA for repeated measures with Dunnett or Bonferroni post-tests using the Graph Pad Prism 6 Software (Prism, San Diego, CA) was used for statistical calculations. Clinical data was analyzed by using Kruskal Wallis followed by Mann Whitney. P ≤ 0.05 was considered statistically significant.

## Additional Information

**How to cite this article**: Baker, J. R. *et al*. Oxidative stress dependent microRNA-34a activation via PI3Kα reduces the expression of sirtuin-1 and sirtuin-6 in epithelial cells. *Sci. Rep.* **6**, 35871; doi: 10.1038/srep35871 (2016).

## Supplementary Material

Supplementary Information
