# Supplementary material for: Oxidative stress dependent microRNA-34a activation via PI3Kα reduces the expression of sirtuin-1 and sirtuin-6 in epithelial cells
Source: Sci Rep. 2016 Oct 21;6:35871. doi: 10.1038/srep35871 (PMC5073335; doi:10.1038/srep35871)
Supplement: Supplementary Information [file srep35871-s1.pdf]

Title: Oxidative stress dependent microRNA-34a activation via PI3Kα reduces the expression of sirtuin-1 and sirtuin-6 in epithelial cells

Authors: J. R. Baker[1], C. Vuppusetty[1], T. Colley[1], Andriana I Papaioannou[2], P. Fenwick[1], Louise Donnelly[1], K. Ito[1] and P. J. Barnes[1*]

Affiliations:
[1] Airway Disease Section, National Heart and Lung Institute, Imperial College, London SW3 6LY, U.K.
[2] 3rd Respiratory Medicine Department, Sismanogleio Hospital, Marousi Athens Greece

* To whom correspondence should be addressed: p.j.barnes@imperial.ac.uk

The authors have declared that no conflict of interest exists

Supplementary Materials:

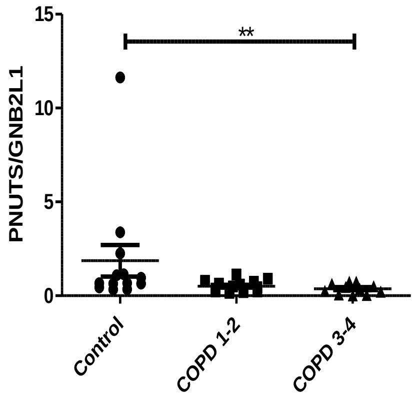

Supplementary Figure. 1. COPD severity is important in the regulation of PNUTS gene expression Lung tissue from resections were obtained from 4 healthy volunteers and 9 non-COPD smoker volunteers (Controls), 15 mild COPD G1-2 (Gold 1 plus Gold 2) and 11 severe COPD G3-4 (Gold 3 plus Gold 4) (COPD) and RNA was extracted. PNUTS levels, normalized to GNB2L1, were examine in lung samples from healthy non-smokers, smokers and COPD. Data are means ± SEM ** P<0.01.

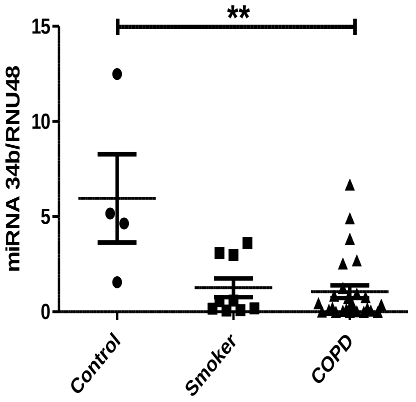

Supplementary Figure. 2. Smoking may regulate the expression of miRNA 34b Lung tissue from resections were obtained from 4 healthy volunteers and 9 non-COPD smoker volunteers (Controls), 15 mild COPD (Gold 1 plus Gold 2) and 11 severe COPD (Gold 3 plus Gold 4) (COPD) and RNA was extracted. MiR-34b levels, normalized to RNU48, were examine in lung samples from healthy non-smokers, smokers and COPD. Data are means ± SEM ** P<0.01.

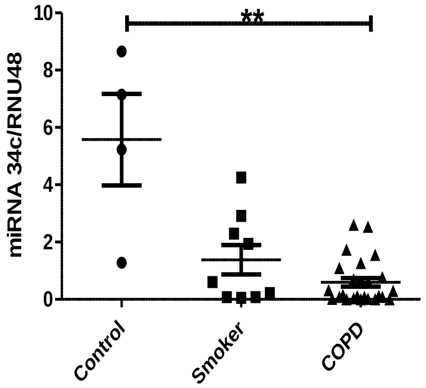

Supplementary Figure. 3. Smoking may regulate the expression of miRNA 34c Lung tissue from resections were obtained from 4 healthy volunteers and 9 non-COPD smoker volunteers (Controls), 15 mild COPD (Gold 1 plus Gold 2) and 11 severe COPD (Gold 3 plus Gold 4) (COPD) and RNA was extracted. MiR-34c levels, normalized to RNU48, were examine in lung samples from healthy non-smokers, smokers and COPD. Data are means ± SEM ** P<0.01.

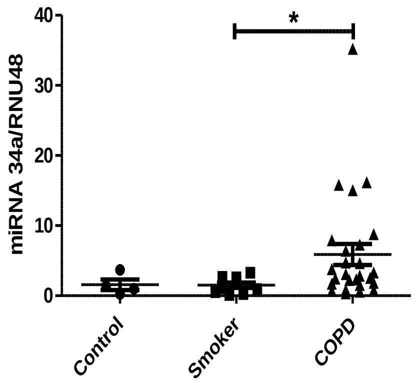

Supplementary Figure. 4. Smoking appears not to regulate the expression of miRNA 34a Lung tissue from resections were obtained from 4 healthy volunteers and 9 non-COPD smoker volunteers (Controls), 15 mild COPD (Gold 1 plus Gold 2) and 11 severe COPD (Gold 3 plus Gold 4) (COPD) and RNA was extracted. MiR-34a levels, normalized to RNU48, were examine in lung samples from healthy non-smokers, smokers and COPD. Data are means ± SEM * P<0.05

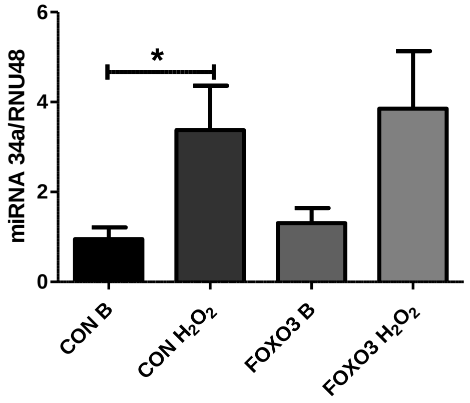

Supplementary Figure. 5. FOXO3a does not regulate the expression of miRNA 34a BEAS2B cells were transfected with either siRNA against FOXO3a or RNA control and then left untreated for 48 hours. MiR-34a expression was detected by quantitative RT-PCR using commercially available TaqMan primers and normalized to expression of RNU48 Data are means ± SEM * $P<0.05$ (n=4).

Tables

Table 1. The characteristics of study subjects for peripheral lung tissues.

	Non-smoker	Smokers without COPD	COPD1	COPD2	COPD3	COPD4
Number (M/F)	4 (1/3)	9 (3/6)	7 (5/2)	8 (3/5)	4 (3/1)	7 (3/4)
Age (yr)	70.5±6.1	61.7 ± 12.4	67.7 ± 6.9	63.0 ± 9.3	63.3 ± 29.3	59.6 ± 4.5
Pack-years	N/A	46 ± 32.9	44.3 ± 16.8	61.2 ± 8.4	46.7 ± 21.8	40.5 ± 18.8
FEV_1 (L)	2.2 ± 0.6	2.74 ± 0.65	2.74 ± 0.55	1.86 ± 0.36	1.76 ± 0.75$^{\#}$	0.52 ± 0.19$^{\#\#}$
FEV_1 (%)	97.4 ± 14.5	100.5 ± 15.3	89.1 ± 3.8	65.3 ± 18.8$^{\#}$	48.8 ± 21.4$^{\#\#}$	16.5 ± 6.7$^{\#\#\#}$
FEV_1 / FVC	80.4 ± 5.5	75.1 ± 4.5	64.3 ± 3.8 $^{\#\#}$	61.2 ± 8.4$^{\#\#}$	49.5 ± 21.8$^{\#\#}$	27.7 ± 10.9$^{\#\#\#}$

COPD patients were categorized by GOLD severity.
Abbreviations: COPD = chronic obstructive pulmonary disease; pack-year = {number of cigarettes smoked per day / 20 × duration of smoking; FVC = forced vital capacity; FEV_1 = forced expiratory volume in one second; $^{\#}$ p <0.05, $^{\#\#}$ p <0.01 vs. Non-Smoker. Data are expressed as mean value ± standard deviation.

Table 2. The characteristics of study subjects for sputum samples.

	Non-smoker	Smokers without COPD	COPD1	COPD2	COPD3	COPD4
Number (M/F)	1(1/0)	4 (3/1)	2 (2/0)	3 (3/0)	6 (4/2)	2 (2/0)
Age (yr)	65	65.5 ± 8.2	56 ± 7.1	72.0 ± 2.8	69.7 ± 6.3	70.5 ± 12.0
Pack-years	N/A	21.3 ± 8.5	50 ± 0	101.7 ± 85.5	60.3 ± (40.6)	102.5 ± 45.9
FEV_1 (L)	2.3	2.59 ± 0.72	3.42 ± 0.2	1.71 ± 0.24	0.97 ± 0.25	0.73 ± 0.07
FEV_1 (%)	92	99.25 ± 23.6	92.5 ± 4.95	58.7 ± 6.6	35.9 ± 3.9	23.5 ± 2.1
FEV_1 / FVC	77	82.3 ± 1.8	70 ± 4.3	45.0 ± 11.3	37.4 ± 6.8	31 ± 1.41

COPD patients were categorized by GOLD severity.
Abbreviations: COPD = chronic obstructive pulmonary disease; pack-year = {number of cigarettes smoked per day / 20 × duration of smoking; FVC = forced vital capacity; FEV_1 = forced expiratory volume in one second Data are expressed as mean value ± standard deviation.

Table 3. The characteristics of study subjects for primary epithelial cells.

	COPD
Number (M/F)	7 (6/1)
Age (yr)	67.3 ± 10.2
Pack-years	41.5 ± 16.9
FEV_1 (L)	1.85 ± 0.92
FEV_1 (%)	71.2 ± 27.9
FEV_1 / FVC	49.1 ± 13.5

COPD patients were categorized by definition of GOLD.
Abbreviations: COPD = chronic obstructive pulmonary disease; pack-year = {number of cigarettes smoked per day / 20 × duration of smoking;; FVC = forced vital capacity; FEV_1 = forced expiratory volume in one second. Data are expressed as mean value ± standard deviation.
